# *Apolipoprotein E ε4* Allele was Associated With Nonlesional Mesial Temporal Lobe Epilepsy in Han Chinese Population

**DOI:** 10.1097/MD.0000000000002894

**Published:** 2016-03-07

**Authors:** Zhimei Li, Chengyun Ding, Xiping Gong, Xiaofei Wang, Tao Cui

**Affiliations:** From the Department of Neurology, Beijing Tiantan Hospital, Capital Medical University, China National Clinical Research Center for Neurological Diseases (ZL, XG, TC); Department of Neurology, The 301 PLG General Hospital (CD); and Department of Sociology (XW), Peking University, Beijing, China.

## Abstract

Apolipoprotein E (*APOE*) gene has been implicated as one of the genes susceptible to temporal lobe epilepsy (TLE), but the association is inconsistent. We carried out a study to investigate the association of *APOEε4* allele with a subtype of TLE-nonlesional mesial temporal lobe epilepsy (NLMTLE) in Han Chinese people.

The study consisted of total 308 NLMTLE patients and 302 controls in Han Chinese. The *APOE* polymorphisms were genotyped using polymerase chain reaction (PCR) DNA sequencing. We compared the frequency of *APOEε4* allele and carrying status between NLMTLE patients and control subjects to test for the association of *APOEε4* allele with NLMTLE clinical status.

Carrying status of *APOEε4* allele was significantly associated with the risk of NLMTLE. No effect of *APOEε4* allele was found on the age of onset, duration of epilepsy, or frequency of seizure. Moreover, there was no association between *APOEε4* allele and hippocampal sclerosis (HS) or febrile convulsion (FC) history.

Our study provided an evidence that *APOEε4* allele was a possible risk factor for NLMTLE, and further study with a larger sample is needed to warrant this finding.

## INTRODUCTION

Temporal lobe epilepsy (TLE) is the most common form of partial epilepsy with a variety of clinical manifestations and underlying etiology.^1^ It is multifactorial and affected by both genetic and environmental factors.^2,3^ In recent years, growing evidence has indicated that genetic predisposition is an important risk factor of TLE.^4,5^ Although some patients of TLE are found in large pedigree and in Mendelian form,^6^ most patients with TLE are sporadic. Therefore, susceptibility genes may play some important roles in the pathogenesis, especially may be involved in specific aspects of clinical phenotypes and pathological characteristics, or through gene–gene interactions.

Apolipoprotein E (APOE) is a critical protein for the maintenance and repair of injured cell membrane, as well as neurite growth, dendritic remodeling, and synaptogenesis.^7^The *APOE* gene expression is upregulated after brain damage from various causes.^8^ There are 3 allelotypes *ε2*, *ε3*, and *ε4* in *APOE*. In the presence of *APOEε4* allele, brain's ability to repair and to form new synapses is impaired.^9^ The link between *APOE* polymorphisms, especially *APOEε4* allele, and TLE have recently drawn great attention.^10^

Recent studies indicated that *APOEε4* allele was related to the phenotypic severity of TLE such as early onset of habitual seizures, increased risk of verbal learning deficit, longstanding seizures which affect memory performance and increased risk of postictal confusion.^10–14^ However, some other studies did not find the association between *APOE* polymorphisms and TLE.^15–18^ TLE can be classified into mesial TLE (MTLE) and lateral TLE (LTLE) by the origin of seizure or into lesional and nonlesional by etiology. Nonlesional mesial temporal lobe epilepsy (NLMTLE) is a unique form of TLE because of its resistance to drug therapy.^19^ A better understanding of the molecular role of *APOE* in the development of NLMTLE may help to understand the pathogenic mechanism and to develop antiepileptogenic therapies. Here, we reported a study to examine the association of *APOEε4* allele with the specific NLMTLE in Han Chinese population.

## METHODS

### Subjects

This study was reviewed and approved by the Ethics Committee of Beijing Tiantan Hospital, Capital Medical University in China in accordance with the Declaration of Helsinki. Informed consent was obtained from each subject enrolled.

This study consisted of 308 patients with NLMTLE and 302 healthy controls. All patients were those who visited and diagnosed at the clinics at Epilepsy Center in Beijing Tiantan Hospital, Capital Medical University from June 2005 to January 2010. Diagnosis of NLMTLE was made based on the clinical history, electroencephalogram, and magnetic resonance imaging (MRI). The inclusion criteria included: seizure semiology consistent with MTLE; anterior and mesial temporal ictal or interictal discharges, without generalized or extra temporal discharges; no lesion such as cerebral tumor, cortical dysgenesis, vascular lesion or malformation, or posttraumatic scar other than atrophy and increased signal in hippocampal formation identified by MRI; and without epilepsy family history. In this study, MRI was performed by using sequences and slices designed to optimize visual detection of hippocampal sclerosis (HS), following the protocol described by Kanemoto et al.^20^ Unrelated healthy subjects were recruited from individuals who came for routine physical examination at the clinics of Beijing Tiantan Hospital, Capital Medical University from June 2005 to January 2010, and subjects without family history of neurological diseases were randomly selected as controls. Both NLMTLE patients and controls were of Han Chinese ancestry and did not have a history of hypertension, hyperlipidemia, and diabetes.

## GENOTYPING

*APOE* polymorphisms were genotyped using polymerase chain reaction (PCR)-DNA sequencing. Venous blood was drawn from each individual and stored at −80 °C. Genomic DNA was extracted from whole blood using standard method. A 292-bp PCR fragment spanning *APOE* polymorphisms was amplified by using the following primers: 5′-AAC AAC TGA CCC CGG TGG CG-3′; 5′-ATG GCG CTG AGG CCG CGC TC-3′. PCR conditions used for amplification were 94 °C for 8 minutes, followed by 35 cycles of 94 °C for 45 seconds, 65 °C for 45 seconds, and 72 °C for 45 seconds. The final extension step at 72 °C was lengthened to 8 minutes. PCR products were tested by 1.2% Agarose Gel Electrophoresis and the products were purified by DNA rapid purification kit (Beijing:Dingguo Biotech, China). DNA sequencing PCR reaction mixture (5 μL) consisted of templates DNA 2 μL, sequencase 2 μL, and primer 1 μL (5′AAC AAC TGA CCC CGG TGG CG 3′). PCR conditions used for sequence were 95 °C for 2 minutes, followed by 35 cycles of 95 °C for 3 seconds, 50 °C for 3 seconds, and 60 °C for 90 seconds. The final extension step at 60 °C was lengthened to 5 minutes. The products were then sequenced using an ABI 3730 × L DNA Sequencer (Perkin-Elmer Applied Biosystems, Norwalk, CT).

### Statistical Analysis

The Stata statistical software Windows Release 12.0 was used for statistical data analysis. Hardy-Weinberg equilibrium of *APOE* polymorphisms was assessed using χ^2^ test. Tests for differences in frequency of allele, carrier of *APOEε4*, and genotype by disease status and by gender were performed using the χ^2^ test or Fisher exact test when appropriate. Mann–Whitney *U*-test was used for quantitative clinical variables as variance of some variables were slightly different by *APOEε4* carrying status, and normality checking was not appropriate due to smaller sample size in *APOEε4* carriers; Chi-square tests were used in comparison of qualitative clinical variables. Multiple logistic regression was used to test for the association of *APOE* allele with the risk of NLMTLE.

## RESULTS

At the time of the study the mean age of the NLMTLE patients was 29.6 ± 11.5 years and the mean age of control subjects was 30.7 ± 9.0 years (*P* = 0.099). The patient group consisted of 184 males and 124 females, whereas the control group included 165 males and 137 females (*P* = 0.203). There was no significant difference in mean age and sex ratio between NLMTLE patient group and control group. In the patient group, the age of seizure onset was 19.9 ± 11.8 years, duration of epilepsy was 9.5 ± 8.0 years, and frequency of seizure was 5.7 ± 4.2 times/month (Table 4). Based on the imaging of MRI, 184 NLMTLE patients were diagnosed with HS and 124 patients were diagnosed without HS. Of the patients, 88 patients had a history of febrile convulsion (FC).

**TABLE 4:**
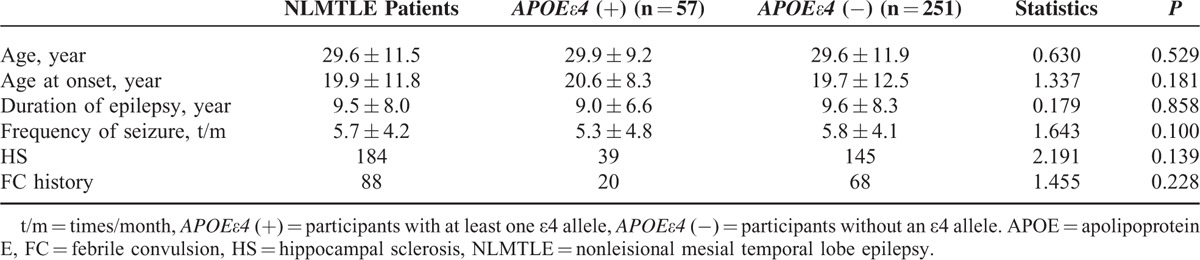
Clinical Variables of NLMTLE Patients by Carrying Status of *APOEε4* Allele

The frequency of genotype in both patients and controls were in Hardy–Weinberg equilibrium. The frequency of *APOE* genotype was presented in Table 1. Overall, the frequency of *APOE* genotype was 0.66%, 11.80%, 0.66%, 71.97%, 14.92%, and 0.00% for ε2/2, ε2/3, ε2/4, ε3/3, ε3/4, and ε4/4, respectively. There were no difference in the frequency of *APOE* genotype between NLMTLE patients and controls (χ^2^ = 6.413 *P* = 0.170), likely due to the sparse sample of limited sample size in some genotypes. When classifying the sample subjects by *APOEε4* allele carrying status (Table 2), we found that 18.51% of NLMTLE patients carrying *APOEε4* allele, which was higher than in controls (12.58%). The difference in the *APOEε4* carrying status was statistically significant (χ^2^ = 4.0695, *P* = 0.04366 < 0.05). When adjusted for sex and age in multiple logistic regression analysis, carrying of *APOEε4* allele was significantly associated with NLMTLE disease (Table 3) and the effect size was moderate to large (OR = 1.581, 95% confidence interval 1.012–2.471, *P* = 0.044). In the NLMTLE patient group, we did not observe any significant difference in clinical variable between *APOEε4* allele carriers and noncarriers (Table 4). The age at onset of seizure (*P* = 0.181), duration of epilepsy (*P* = 0.858), and frequency of seizure (*P* = 0.100) did not differ in NLMTLE patients by *APOEε4* allele carrying status, and neither was different for the presence of HS (*P* = 0.139) and FC history (*P* = 0.228).

**TABLE 1:**
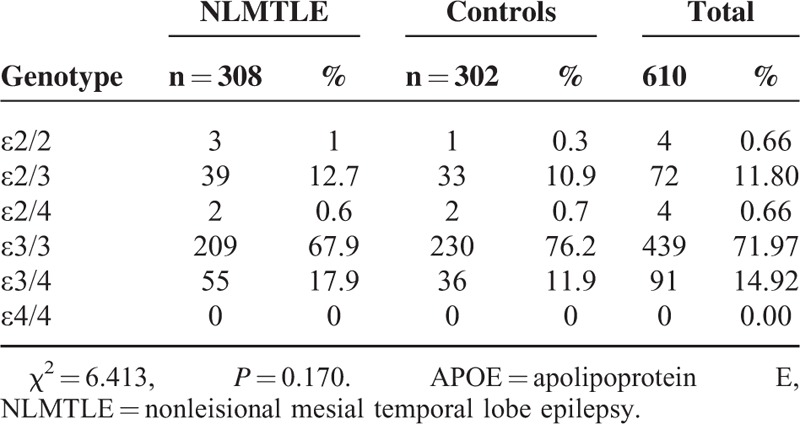
Frequency of *APOE* Genotype in the Study Sample and by NLMTLE Status

**TABLE 2:**
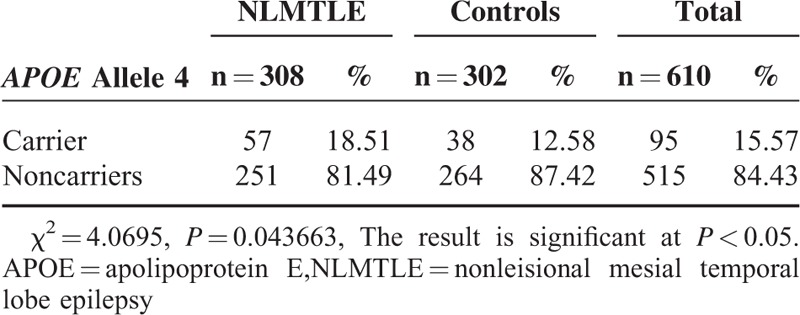
Carrying of *APOEε4* Allele by NLMTLE Disease Status

**TABLE 3:**
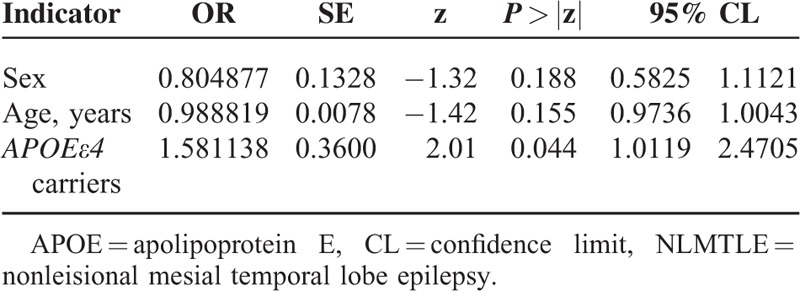
Multiple Logistic Regression Estimates of *APOEε4* Allele on NLMTLE Disease Status

## DISCUSSION

In this study, we examined the association of *APOE* polymorphisms with NLMTLE disease status in Han Chinese population. Although there was not significant association of NLMTLE with *APOE* genotype likely due to limited sample size, we found that carrying of *APOEε4* significantly increased the risk of NLMTLE in Han Chinese population. The proportion of *APOEε4* carriers in controls was 12.58%, which was consistent with that in other studies, suggesting that our controls were representative in Han Chinese population. To our knowledge, this is the first report on the association between *ApoEε4* and NLMTLE in Han Chinese population.

Our finding was consistent with previous studies that showed *APOE* as a potential susceptibility gene to TLE. Animal study has shown that brain's ability to repair damage and form new synapses was impaired in presence of *APOEε4* allele.^21^ The *APOEε4* allele promotes the intracerebral accumulations of β-amyloid, which plays a central role in increasing neuronal susceptibility to damage.^22^ Studies on expression and presence of β-amyloid precursor protein and β-amyloid have been performed and demonstrated a link between *APOEε4* allele and TLE using temporal lobe specimens.^23–25^ Further studies found the *APOEε4* allele might play an active role in modulating some clinical features of TLE. *APOEε4* allele was associated with earlier onset of TLE in a meta-analysis of 7 published studies, carriers of *APOEε4* allele tended to have 5 years earlier than noncarriers in onset of the disease,^10,26^ risk of posttraumatic seizures,^11^ refractory complex partial seizures,^27^ verbal learning deficit,^12^ memory loss,^13^ and postictal confusion.^14^ However, there are other studies that failed to find a significant association between TLE and *APOE* polymorphisms in several populations including Han Chinese people.^18,28^

It is important to emphasize that in our study the proportion of each *APOE* genotype or allele in both the controls and patients is very similar to that previously found in the general population in Han Chinese people.^29–31^ TLE is one of complex human disorders that are clinically and genetically heterogeneous. The pathogenic effect of each putative “susceptibility gene” is small or modest, and environmental factors may play a part role in the development of TLE. The related studies on *APOE* and TLE also showed inconsistent results, probably due to the clinical heterogeneity of the patients sample. According to the focus of seizure origins, TLE can be classified into MTLE and LTLE subtype. The clinical and pathological feature may be different between MTLE and LTLE. As for the etiology MTLE is further classified into leisonal and nonleisonal subtype. HS is the most common histopathologic form of nonleisonal MTLE. Although the pathomechanism of HS is unclear, some studies show that early precipitant events such as febrile convulsion are correlated with HS. This indicates that susceptibility gene may play a role in the development of HS and MTLE. Genetic study of pure type of nonlesional MTLE alone may be helpful for identifying risk variants. We have seen that previous study^18^ in Han Chinese population has mixed all of patient samples with different subtypes such as nonlesional TLE and developmental lesions, therefore not comparable with our observations.

Association studies seem to be a common approach to identify genes associated with complex human diseases such as TLE. However, we are aware of those limitations such as sample size, selection bias, etc.^32^ It should be noted that our study fulfill the fundamental requisites and has minimal of those limitations. First, as the frequency of *APOE* allele varies among different ethnic populations, control subjects were carefully matched for ethnic background in our study. Second, it was reported that the frequency of *ApoEε4* allele in general Han Chinese was 3.5% to 12.9%, which was consistent with that in our study sample. In the meantime, the proportion of *APOEε4* allele carrying was 12.58% in controls, which was close to population-based sample in multiple ethnic group including Han Chinese (14.25%).^30,31^ Our sample size provided enough statistical power (80%) to detect an allelic association with an modest effect size (OR = 2.0) for a 10% probability of exposure in controls at a level of 0.05. In addition, the association may have arisen by chance, or may be artifactual because of the weaknesses of study design and selection bias.^33,34^ Focusing on subtype of TLE such as NLMTLE may reduce the disturbance of possible underlying etiology. Although we provided an evidence that the *APOEε4* allele was a risk factor for NLMTLE, we did not find any significant association of *APOEε4* allele carrying status with the age at onset of seizures, duration of epilepsy, frequency of seizure, HS, and FC history in NLMTLE patients. It is worth noting that there were some trends of association with age at onset of seizure, frequency of seizure. A further study with a larger cohort of patients is needed. Our study illustrated *APOE* might be a possible susceptibility gene for NLMTLE. Further studies in independent population samples should be investigated to confirm the association; and molecular study is also needed to understand the mechanism of this genetic association in the future.
